# Functional mapping of sensorimotor activation in the human thalamus at 9.4 Tesla

**DOI:** 10.3389/fnins.2023.1116002

**Published:** 2023-03-15

**Authors:** Edyta Charyasz, Rahel Heule, Francesko Molla, Michael Erb, Vinod Jangir Kumar, Wolfgang Grodd, Klaus Scheffler, Jonas Bause

**Affiliations:** ^1^Department of Biomedical Magnetic Resonance, University of Tübingen, Tübingen, Germany; ^2^Department for High Field Magnetic Resonance, Max Planck Institute for Biological Cybernetics, Tübingen, Germany; ^3^Graduate Training Centre of Neuroscience, Tübingen, Germany; ^4^Center for MR Research, University Children’s Hospital, Zurich, Switzerland; ^5^Center for Neurology, Hertie-Institute for Clinical Brain Research, Tübingen, Germany

**Keywords:** fMRI, thalamus, ultra-high field fMRI, high-resolution imaging, thalamic nuclei, sensorimotor, tactile, motor

## Abstract

Although the thalamus is perceived as a passive relay station for almost all sensory signals, the function of individual thalamic nuclei remains unresolved. In the present study, we aimed to identify the sensorimotor nuclei of the thalamus in humans using task-based fMRI at a field strength of 9.4T by assessing the individual subject-specific sensorimotor BOLD response during a combined active motor (finger-tapping) and passive sensory (tactile-finger) stimulation. We demonstrate that both tasks increase BOLD signal response in the lateral nuclei group (VPL, VA, VLa, and VLp), and in the pulvinar nuclei group (PuA, PuM, and PuL). Finger-tapping stimuli evokes a stronger BOLD response compared to the tactile stimuli, and additionally engages the intralaminar nuclei group (CM and Pf). In addition, our results demonstrate reproducible thalamic nuclei activation during motor and tactile stimuli. This work provides important insight into understanding the function of individual thalamic nuclei in processing various input signals and corroborates the benefits of using ultra-high-field MR scanners for functional imaging of fine-scale deeply located brain structures.

## Introduction

Sensorimotor processing engages cortical and subcortical areas. The extensive sensorimotor investigations implicate the motor cortex of the frontal lobe, the sensory cortex of the parietal lobe, and the cerebellum (Grodd et al., 2001; Hermes et al., 2012; Siero et al., 2014; Filippi et al., 2016). The sensorimotor pathway also engages the thalamus (Mo and Sherman, 2019).

The thalamus has been shown to be involved in sensorimotor processing in monkeys (Benevento et al., 1977; Darian-Smith et al., 1990), mice (Wang et al., 2021), and rats (Petersen, 2019). So far, sensorimotor functional mapping in the thalamus is under-investigated in the human brain. The thalamus encompasses small motor and sensory nuclei, and therefore requires high-resolution investigations. In this study, we aim at utilizing the power of ultra-high field fMRI to investigate the individual thalamus’s functional response during sensorimotor task at the single-subject level. Such high-resolution sensorimotor mapping within the thalamus may help better strategize the potential therapeutic planning and understanding of sensorimotor dysfunctions. Assessing the individual subject-specific fMRI responses allows perceiving the overall sensorimotor experience of the participating subjects to the employed stimuli and analyzing the intersubject variability, which may arise due to anatomical or functional differences across the investigated population.

The thalamus is a paramedian symmetrical mass of gray matter within the vertebrate brain that arises during embryonic development as the main constituent of the diencephalon and attaches to the upper part of the brainstem through the telencephalon. Anatomically, the thalamus is divided in four major groups (the anterior, medial, lateral, and the posterior group), which can further be functionally distinguished into first-order and higher-order nuclei based on the origin of their driving inputs (Haber and McFarland, 2001; Sherman and Guillery, 2002; Theyel et al., 2010; Lambert et al., 2017). First-order nuclei receive driver signals from peripheral sources and project to the cerebral cortex. For example, thalamic motor nuclei such as the ventral anterior (VA) and ventral lateral (VL) receive primary afferents from the basal ganglia as well as the cerebellum and send efferents to the premotor as well as primary motor cortices (Guillery and Sherman, 2002; Bosch-Bouju et al., 2013; Gaidica et al., 2018). Higher-order nuclei, such as the pulvinar (Pu) and the mediodorsal (MD), receive their driving input from layer 5 of the cortex and participate in cortico-thalamo-cortical (or transthalamic) circuits in projecting them back to the cortex (Wurtz et al., 2005; Saalmann, 2014). While the sensorimotor involvement of the cortical areas and the cerebellum has been extensively studied for decades in humans as well as in primates (Logothetis et al., 2001; Kishore et al., 2014; Wang et al., 2018; Edwards et al., 2019) using functional magnetic resonance imaging (fMRI) (Rao et al., 1995; Lotze et al., 2000; Rosazza et al., 2014; Landelle et al., 2021), electroencephalography (EEG) (Muthukumaraswamy and Johnson, 2004; Morash et al., 2008; Zhao et al., 2019), magnetoencephalography (MEG) (Sebastiani et al., 2014), and electrophysiology (Arce-McShane et al., 2016; Ranieri et al., 2022), the human thalamus has widely remained inaccessible. Due to its central location within the brain, its small size, and the lack of sufficient contrast in various imaging modalities, the delineation of thalamic nuclei using fMRI remains challenging. Ongoing progress in the development of accelerated MR acquisition techniques, such as parallel imaging or multi-band radiofrequency (RF) pulses combined with adapted RF coil designs to maximize the achievable signal-to-noise-ratio (SNR), yields increased spatial resolution while maintaining high temporal resolution. Nevertheless, a reliable localization of thalamic nuclei and their functional activation is yet limited by insufficient SNR and consequently spatial resolution constraints at conventional clinical field strengths (≤ 3 Tesla).

Consequently, in humans, the involvement of the thalamus in sensory and motor processing has only been vaguely characterized regarding the precise anatomical location and functional contribution of the various thalamic nuclei. To obtain the specific functional activation of the thalamic nuclei involved in sensorimotor and other functional processing in a robust and reproducible manner, the use of ultra-high field MR scanners may prove beneficial due to their increased SNR, enhancing spatial and temporal resolution as well as image contrast (Pohmann et al., 2011, 2016).

Thus, functional MRI at ultra-high field strength may provide an opportunity to investigate the blood oxygen level-dependent (BOLD) activations of specifically involved thalamic nuclei in sensorimotor processing. In this study, the functional involvement of thalamic nuclei during finger movement and tactile finger stimulation in humans was examined for the first time using an ultra-high field strength of 9.4 Tesla. Due to the challenges of accurately identifying and segmenting the individual fine-scale thalamic nuclei in each subject, this proof-of-principle study focused on a single-subject rather than a group analysis, which enabled to preserve spatial specificity. The results of this study may contribute to depict a more comprehensive view of the differential role of the thalamus in the context of motor and sensory processing as well as to enhance our understanding of the functional architecture of the thalamus. In addition, ultra-high field fMRI may allow a refined investigation of the role of the thalamus in neurological motor disorders like Parkinson’s disease, dystonia, and essential tremor.

## Materials and methods

### Subjects

Ten healthy right-handed adults with normal or corrected to normal vision and a mean age of 27 years (range 21–34 years; five females) participated in the study. The study was approved by the local research ethics committee and all participants gave written informed consent prior to participation.

### Experimental design and setup

All participants performed two fMRI block design tasks with the right hand: tactile-finger task and finger-tapping task during a single session. The left hand was not engaged during any experiments of this study.

For both task, the output of a trigger pulse prior to the acquisition of each volume repetition time (TR) was implemented into the MR sequence to synchronize the fMRI paradigm with the MR acquisition. The tasks were projected onto a translucent screen using a projector. The participants viewed the stimuli *via* a mirror attached to the head coil. Before the main experiment, participants performed one complete training run outside the scanner.

The tactile stimulation system used a pneumatically controlled setup to deliver precise and controlled air pressure pulses to the participant’s fingertip. The custom-built electronic control circuit, connected to the air compressor and plastic tubes, was interfaced with a stimulus computer through the digital controller (mbed, LPC1768). The tubes conveyed air pressure (2.5 bar) to the pneumatic stimulus finger clips (MEG International Services Ltd., Coquitlam, Canada). The timing of the tactile stimuli was controlled using Matlab 2019b (The MathWorks, Inc., Natick, MA, USA) and the PSYCHTOOLBOX (Brainard, 1997; Pelli, 1997). The presentation and timing of the finger-tapping stimuli was controlled by Presentation^®^ software (Version 18.0, Neurobehavioral Systems, Inc., Berkeley, CA)^1^.

#### Tactile-finger task

The tactile stimulation was delivered concomitantly to the fingertips of the thumb (D1), index finger (D2), middle finger (D3), and ring finger (D4) in the form of air pulses through an inflatable finger clip. Each single air pulse caused deviation of the pneumatic membrane (approximately 40 mm^2^) toward the skin surface by a pulse of pressed air at 2.5 bar for a duration of 250 ms. Stimulation pulses (ON-phase) were delivered every second (1 Hz) in blocks of 20 s to all four fingers, as specified above, followed by a rest period (OFF-phase) of 20 s. Data were acquired during seven runs of 12 cycles each. The stimulation was carefully timed to begin at the acquisition of the first volume of each block, following the initial trigger. To focus the subjects’ attention on the stimulation and to prevent habituation, a random number of stimulation pulses (between zero and four per stimulation block) were skipped, resulting in an average of 210–240 air pulses per fingertip. The particular number of the pulses and the time at which these pulses were skipped was chosen randomly for each block. Subjects had to report to the experimenter the total number of blocks with missing pulses in the break between stimulation sessions. In addition, participants were instructed to focus on a fixation cross presented on the screen during the experiment. Two of the ten subjects (S9 and S10) could not perform the tactile task properly due to technical issues and were excluded from the subsequent analysis of those data.

#### Finger-tapping task

The task paradigm consisted of 12 visually cued cycles (each with a total duration of 41 s) divided into alternating finger tapping blocks (20 s) and rest blocks (20 s). Before each movement block, a 1 s interval was used to allow subjects to get ready for the task. Participants were instructed to tap their right fingers in an ordered fashion [index finger (D2), middle finger (D3), ring finger (D4), and little finger (D5), respectively] against the thumb (D1). The tapping rate was paced with a visual cue (blinking arrow). The tapping frequency was approximately 2.5 Hz. Images were collected during one run. All subjects were able to successfully complete the finger-tapping task.

### MR data acquisition

MRI data were acquired on a 9.4T whole-body MRI scanner (Siemens Healthineers, Erlangen, Germany), using an in-house-built head-coil with 16 transmit and 31 receive channels (Shajan et al., 2014).

Structural imaging: A high-resolution T1-weighted scan was acquired with a magnetization-prepared rapid acquisition gradient echo (MPRAGE) sequence [inversion TR = 3.8 s; TE = 2.50 ms; FA = 6°; FOV = 192 mm; 288 sagittal slices; voxel size = 0.6 × 0.6 × 0.6 mm^3^; GRAPPA acceleration factor (*R*) = 2 × 2; partial Fourier = 6/8] covering the whole brain for anatomical reference. In addition, whole brain structural scans for cortical and thalamic segmentation were collected on a Siemens Healthineers Prisma Fit 3T whole-body MRI scanner. For each subject, high-resolution T1-weighted MPRAGE [inversion TR = 2.4 s; TE = 2.22 s; FA = 8°; FOV = 256 mm; 208 sagittal slices; voxel size = 0.8 × 0.8 × 0.8 mm^3^; GRAPPA acceleration factor (*R*) = 2] and T2-weighted 3D fast spin echo [TR = 3.2 s; TE = 5.63 s; FOV = 256 mm; 208 sagittal slices; voxel size = 0.8 × 0.8 × 0.8 mm^3^; GRAPPA acceleration factor (*R*) = 2] data sets were acquired.

Task-based fMRI: Blood oxygenation level dependent (BOLD) data were collected using a 2D gradient-echo multi-band (MB) echo-planar imaging (EPI) sequence with 86 interleaved slices per volume providing full brain coverage and acquired parallel to the AC - PC line (TR = 2 s; TE = 22 ms; FA = 50°; FOV = 198; voxel size = 1.25 × 1.25 × 1.25 mm^3^, *R* = 4, MB factor = 2, bandwidth = 1666 Hz/Px, and anteroposterior phase encoding). For the purpose of distortion correction, MB-EPI scans with reversed phase encoding direction (posterior-anterior) were performed with otherwise identical parameters as the main experiment.

Functional imaging sessions consisted of seven tactile-finger runs (255 volumes each, acquisition time of 8.5 min), one finger-tapping run (265 volumes, acquisition time of about 9 min) and one run with reversed phase encoding direction for distortion correction (10 volumes) at the end of the session.

Physiological parameters (cardiac pulsation and respiration rate) were recorded during the functional scans using MR compatible devices (Acknowledge, Biopac Systems, Inc., Goleta, CA, USA). Synchronization with the MR sequence was achieved by parallel recording of the sequence trigger signal. However, due to unstable recording by the BIOPAC system during the experiments, complete physiological data could not be collected for three subjects (S3, S4, and S6), and four subjects (S3, S6, S7, and S8) for the motor and tactile task, respectively. Therefore, the successfully recorded physiological data for the remaining individuals were not used in the subsequent analysis.

### MRI data analysis

#### Data preprocessing

The fMRI data preprocessing and analysis of both tasks was conducted by using the SPM12 software (R7771^2^) implemented in MATLAB R2017b (The MathWorks, Inc., Natick, MA, USA). Before analysis, the first five volumes of the functional data of each run were discarded to mitigate T1 saturation effects.

All functional images were first corrected for the acquisition time delay between slices, then spatially realigned to the first image to correct for rigid body motion, and subsequently corrected for thermal noise fluctuations and distortions. Image distortions were corrected by the TopUp tool (Andersson et al., 2003) of the FSL (Smith et al., 2004) library. NORDIC (Moeller et al., 2021) denoising, using PCA to correct for non-Gaussian noise distributions, was applied to the magnitude images in order to reduce thermal noise. The effect of NORDIC denoising is shown in the Supplementary Figure 1. The functional distortion-corrected data sets were co-registered to the anatomical data and finally spatially smoothed using a Gaussian kernel with a full width at half maximum (FWHM) of 2.5 mm.

**FIGURE 1:**
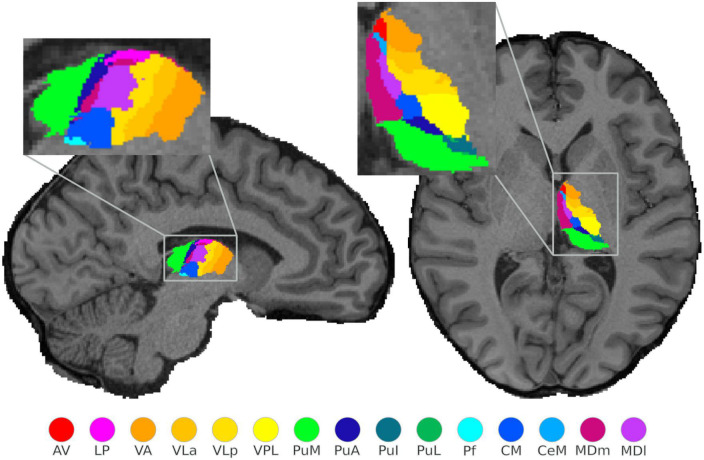
Structural segmentation of the thalamus: illustration of the probabilistically segmented left thalamic nuclei using FreeSurfer in one exemplary subject. The selected sagittal and axial view visualize only 15 nuclei, however, a complete nuclei segmentation of the thalamus was performed.

FreeSurfer software^3^ (version 6.1) (Fischl et al., 1999), incorporating a probabilistic thalamic segmentation algorithm (Iglesias et al., 2018), was used for thalamic segmentations. Our previous findings of unreliability in the application of FreeSurfer to the structural data acquired at 9.4T (0.6 mm isotropic resolution) as a result of the altered image contrast at ultra-high field prompted the use of 3T anatomical data for the purpose of segmentation despite the lower resolution (0.8 mm isotropic). Regions-of-interest (ROIs), for example, whole thalamus, or individual parcellated thalamic nuclei, were extracted as masks from the segmentation, and co-registered to each subject’s anatomical images (cf. Figure 1). All subsequent analyses were carried out in native space. An example of the co-registration of 3T T1-weighted and T2-weighted images to 9.4T T1-weighted data is shown in the Supplementary Figure 2.

#### GLM analysis

For each subject, the general linear model (GLM) approach was used to analyze task-evoked BOLD responses, separately for the tactile and finger-tapping conditions.

For the tactile task consisting of seven runs, these runs were combined into a single GLM analysis. A separate GLM analysis was performed for the finger-tapping task, composed of one run. Task-based statistical parametric t-maps were calculated at the individual subject level using block-style boxcars convolved with a hemodynamic response function (HRF). A temporal high-pass filter with a cut-off of 128 s was applied to correct for low frequency drifts. All six motion parameters (three rotations and three translations) for each run were included as regressors of no interest to correct for residual motion-related variance.

Involvement of single thalamic nuclei was investigated based on the BOLD response to the tactile and motor tasks by using individual whole thalamus masks as explicit masks (at first level statistics).

**FIGURE 2:**
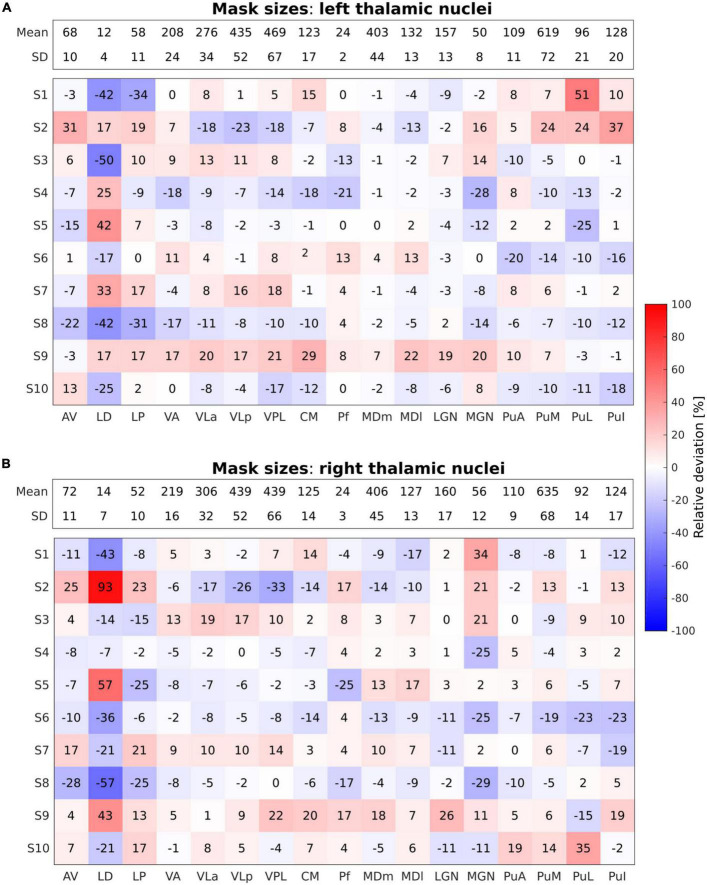
Relative deviation in percentage (%) from the mean mask sizes of the performed segmentations across all individual thalamic nuclei in all subjects for the left **(A)** and the right **(B)** thalamus. The absolute mean mask size pooled over all subjects given as number (#) of voxels along with the respective standard deviation is displayed within the bar on top across all nuclei for both left and right thalamus. Blue and red shades refer to negative and positive relative deviations, i.e., smaller and larger mask sizes as compared to the mean mask size, respectively.

For each of the functional tasks, an MR signal change compared to the rest condition was tested. Individual *t*-maps were obtained with the statistically significant activation threshold set to an uncorrected *p*-value < 0.001 with a minimum cluster size of five voxels, and overlaid onto the individual anatomical image. The minimum cluster size was determined through a consideration of variations in individual thalamus size. To this end, an evaluation of scaling the five voxel threshold was performed by dividing the individual mask size by the mean mask size and then multiplying by 5, resulting in values between 4.6 and 5.6 voxels. Despite this small variability, a minimum cluster size of five voxels was chosen to balance the control of false positive errors and the accurate detection of true effects in the analysis. Thalamic activated clusters were anatomically labeled by overlaying the thresholded activation maps onto the individual thalamic nuclei segmented in FreeSurfer. For the quantification of activation within each thalamic nuclei for the motor and tactile tasks, the number of activated voxels was calculated using the subject–specific thalamic nuclei masks obtained from the thalamic segmentation in FreeSurfer. To preserve the spatial specificity of the individual results and allow the assessment of intersubject functional variability, a group analysis in standard space was not conducted. Additionally, we compared the number of voxels in each thalamic nuclei mask across individual subjects to assess anatomical variability. The count of activated voxels was converted into percentages relative to the total number of voxels in each subject–specific thalamic nuclei mask to normalize for differences in individual thalamic nuclei volumes across subjects. Figure 2 illustrates the relative deviation of individual thalamic nuclei volumes from the mean mask size (mean mask size: pooled over all subjects).

Furthermore, a supplementary whole-brain GLM analysis was performed in a similar manner without utilizing an explicit mask in the first-level statistics. The whole-brain T-statistic maps are presented for four representative subjects in Supplementary Figure 3, with a specific focus on highlighting activation in the cerebral cortex and cerebellum. These results are included in the Supplementary material due to their exploratory nature and secondary role in the study.

#### BOLD response time course

To assess the temporal dynamics of the BOLD signal changes in the thalamic nuclei in response to both tasks and across subjects, time course data from all the activated voxels within each individual thalamic nucleus were extracted for each run (one motor, seven tactile). The percentage change in the BOLD signal was then calculated relative to each run’s rest periods, and the resulting time courses were averaged across activated voxels. Those time courses were subsequently divided into 40 s windows, corresponding to the length of one ON/OFF block, and averaged across runs and twelve blocks for each task type.

## Results

In order to obtain a complete depiction of all thalamic regions activated during the motor and tactile tasks, the activations of the left and the right thalamic nuclei are presented separately. Task-related activations were identified in all subjects who entered the study. As will be presented below, both active and passive tasks evoked consistent activations among subjects. However, tactile-related activations tended to be reduced compared to motor-related activations.

### Motor task

**FIGURE 3:**
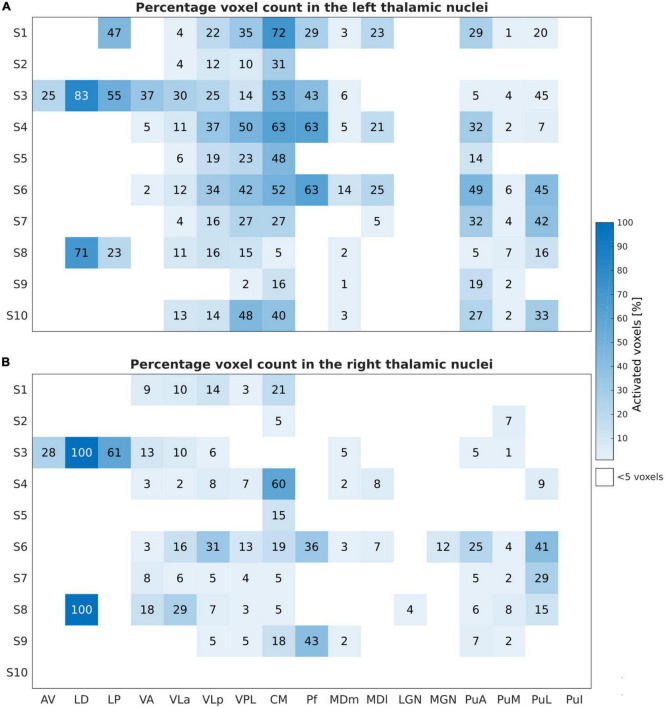
Motor task: Number of activated voxels expressed in percentage (%) relative to the subject-specific mask size during the motor task-based fMRI experiment for the left **(A)** and right **(B)** thalamic nuclei across all subjects and nuclei. These results are obtained by thresholding at an uncorrected *p*-value of < 0.001 and at a minimal cluster size of five voxels within the investigated ROIs.

The percentage of activated voxels during the motor task-based fMRI experiment for the different thalamic nuclei in each subject is summarized in Figure 3 for the left and the right thalamic nuclei.

The finger-tapping task evoked a significant BOLD signal in ten and nine subjects within the left and the right thalamus, respectively, with a clear dominance of the left side. The largest and consistent fraction of activated voxels was found in the left VPL and the intralaminar nucleus CM followed by VLa and VLp of the lateral nuclei group. In addition, we found very consistent activations in the left pulvinar nuclei group (PuA, PuM, and PuL). All subjects showed activations in the left VPL and CM thalamic nuclei. Nine subjects showed activations in the left VLa, and VLp; seven subjects in the left MDm, and four subjects in the left MDl and Pf. Six subjects demonstrated contralateral activations within VLa, VPL, and PuM. Eight and seven subjects showed detectable clusters in the right CM and VLp, respectively. In one subject, S3, the bilateral anterior part of the thalamus (AV and LD nuclei) and the LP nuclei was activated.

**FIGURE 4:**
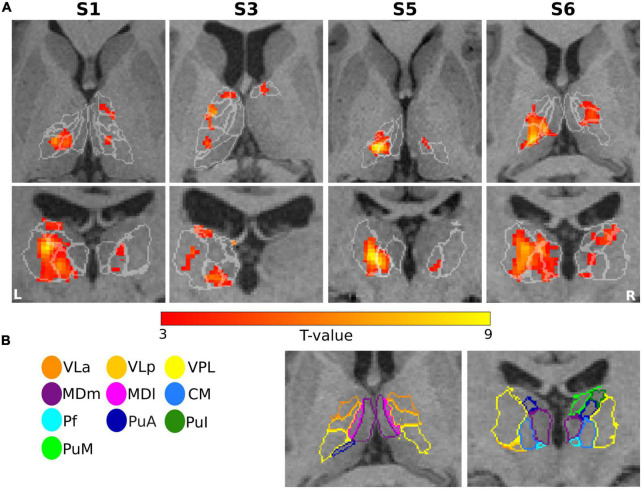
Motor task: **(A)** Results of GLM analysis in the thalamus of four representative subjects (S1, S3, S5, and S6) during the motor task-based fMRI experiment. For each subject, the *t*-contrast maps (*p* < 0.001 uncorrected, and 5-voxel minimum cluster size) are shown, with the individual T1-weighted MPRAGE image (3T) as anatomical underlay. The most representative axial (top) and coronal (bottom) slices oriented in neurological convention (the left side of the image corresponds to the left side of the brain) are depicted for each subject, emphasizing the prominent activation of the thalamic nuclei. **(B)** The contours of the segmented thalamic nuclei are shown for one subject (S6) with the same color-coding as in Figure 1, mapped to the anatomical MPRAGE image.

The first-level GLM analysis results of the finger-tapping task are visualized in Figure 4 for four representative subjects (S1, S3, S5, and S6). All single-subject maps were thresholded at *p* < 0.001 (uncorrected) level and superimposed on their structural images. In summary, the fMRI maps showed dominant activation in the left VPL, CM, and VLp in all representative subjects, with strong activation in the left VLa and the bilateral VA in S3. Bilateral activation was found in MDm and MDl in subject S6.

### Tactile task

**FIGURE 5:**
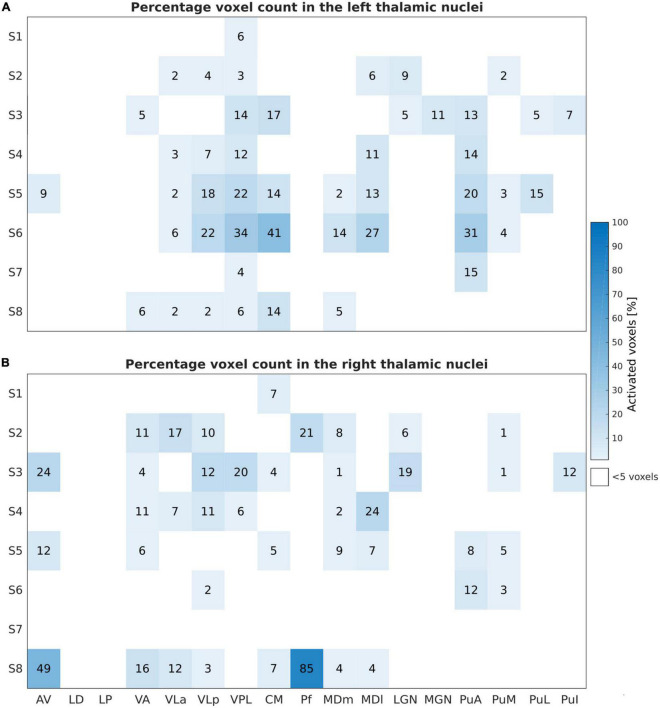
Sensory Task: Activated voxels counts (in percentage) relative to the subject-specific mask size during the tactile task-based fMRI experiment for the left **(A)** and the right **(B)** thalamic nuclei across all subjects and nuclei. Similar to Figure 3, the results are obtained by thresholding at *p* < 0.001, uncorrected, and at a minimal cluster size of five voxels within the investigated ROIs.

**FIGURE 6:**
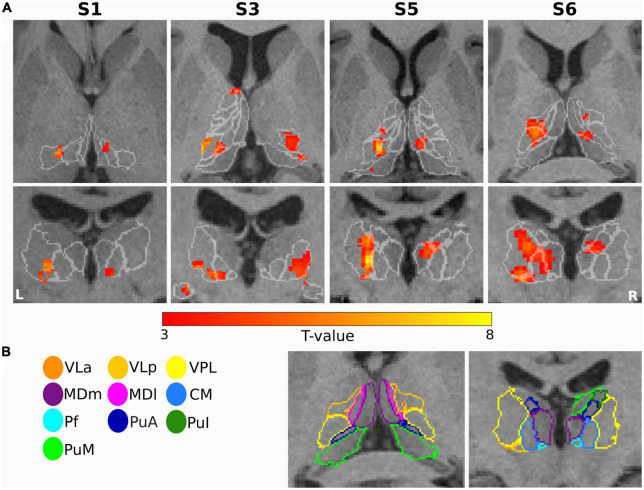
Sensory task: **(A)** Single subject thalamic t-statistic maps (uncorrected *p* < 0.001) obtained for the tactile task-based fMRI experiment. The activation maps (5-voxel minimum cluster size) for subjects S1, S3, S5, and S6 are overlaid on representative axial (top) and coronal (bottom) sections of the 3T T1-weighted MPRAGE image, accentuating the prominent activation of the thalamic nuclei. **(B)** The contours of the nuclei of the thalamus are shown for subject S6 and mapped to the anatomical MPRAGE image.

Figure 5 depicts the percentage of activated voxels during the tactile task-based fMRI experiment within the left and right thalamic nuclei across all subjects, respectively. All subjects except and S7 showed bilateral activation in the thalamus. Consistent activations across all eight remaining subjects were found in the left VPL. By using a threshold of *p* < 0.001 (uncorrected), three and four subjects showed detectable clusters in the left MDm and MDl, respectively. Activation of the left VLa and left VLp nuclei was observed in five out of eight subjects. Bilateral activation was found in CM in four subjects. Like the motor-related activations, the left thalamic nuclei pulvinar region, including PuA, PuM, PuL, and PuI, were activated. No significant activity was found in the bilateral LD, LP and in the left Pf nuclei. Tactile stimulation-evoked BOLD activity in the various thalamic nuclei is shown in Figure 6 representatively for subjects S1, S3, S5, and S6. The single-subject maps (uncorrected *p* < 0.0001) showed consistent bilateral activation of VPL in S3 and ipsilateral activation in VPL, VLp, and CM in S6. Activation of the right AV was found in three subjects (S3, S5, and S8) amongst all measured subjects.

To summarize, at the subject level, we found robust bilateral activation for both stimuli conditions (motor and tactile) in different thalamic nuclei. An individual GLM analysis (*p* < 0.001 uncorrected) comparing the motor task versus the tactile conditions showed that the motor task produced much stronger bilateral activation in the thalamic nuclei, for example, in the CM, VA, VLp, VLa, and PuL, even though just a single run was performed while seven runs were conducted for the tactile stimulus (cf. Supplementary Figure 4).

**FIGURE 7:**
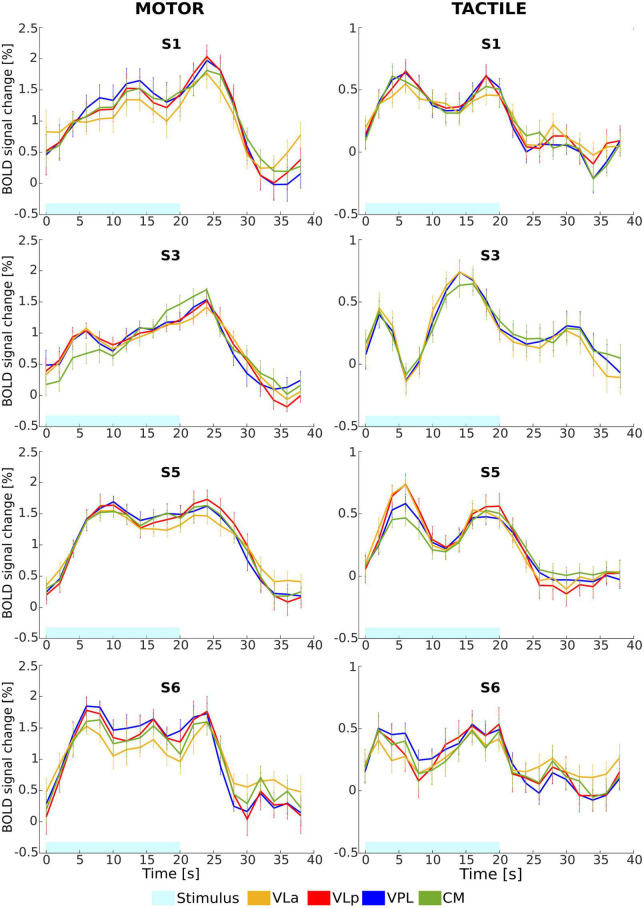
Mean relative BOLD signal change (%) time course from the activated voxels in the left VLa (yellow), VLp (rot), VPL (blue), and CM (green) nuclei in response to motor (left column) and tactile (right column) tasks for four representative subjects (S1, S3, S5, and S6). Error bars represent standard error of the mean.

To further evaluate the difference in response of activated voxels to both tasks, a comparison of the average BOLD signal change time courses from the activated voxels within the thalamic nuclei was performed. The mean relative signal change over 12 block repetitions from the most frequently detected nuclei (left VLa, VLp, VPL, and CM) for four representative subjects are depicted in Figure 7. For the motor task, the averaged BOLD responses measured in the left thalamic nuclei yielded maximum signal changes of 1.7% to 2%. The highest increase in the BOLD signal was observed in the left VPL and CM nuclei, while the lowest increase was seen in the left VLa nucleus across all subjects. The tactile task resulted in a peak of the signal change in the same regions, with maximal values in the range of 0.5 to 0.8%. The observed maximum percent signal changes are notably lower for the tactile task as compared to the motor task, suggesting distinct patterns of neural activation in response to the two tasks. For both tasks, the shape of time courses indicates that, although the temporal patterns of the BOLD responses generally remained consistent within each individual subject across different thalamic nuclei, there is a notable inter-subject variability in the shape of these responses. These results emphasize the agreement between the time course analysis and the GLM analysis in demonstrating stronger activation in the thalamic nuclei in response to the motor task.

## Discussion

In the present study, we investigated the localizability and incidence of activation in the thalamic nuclei, associated with finger-tapping movement and tactile stimulus, at an individual subject level using fMRI at 9.4T. The power of fMRI lies in its ability to resolve fine spatial structures and to detect changes in individual subjects with high spatial specificity, making it superior to other brain imaging modalities (e.g., MEG, EEG, and PET). The accurate delineation of the thalamic nuclei is extremely challenging due to their small sizes, necessitating imaging at ultra-high fields, which are able to provide increased SNR and resolution, in combination with sophisticated segmentation techniques, as well as favoring a single-subject analysis in native space over a group analysis in MNI space to preserve spatial specificity in individual subjects. A subject-specific assessment also avoids the issue of averaging over non-responders and responders in the individual small-sized thalamic nuclei. Therefore, to fully benefit from ultra-high field fMRI and to understand the intersubject variability as a result of potential anatomical and functional differences, we felt that a single-subject analysis is more appropriate for this first in-human study of the functional involvement of the thalamic nuclei in sensorimotor tasks. Furthermore, investigating intersubject variability in healthy volunteers may provide essential insights with respect to future studies in patients with sensory abnormalities and/or motor disabilities (e.g., sensory ataxia, Parkinson’s disease).

Benefiting from the increased SNR at ultra-high field strength, we achieved a spatial resolution (1.25 mm isotropic), which is higher than the one previously reported for fMRI studies in the thalamus (Fischer and Whitney, 2012; Wang et al., 2012; Woodward et al., 2012). In accordance with prior literature pointing to the advantages of small voxel sizes for cortical specificity in BOLD fMRI (Hyde et al., 2001), we successfully detected task-specific activity in individual thalamic nuclei at the employed resolution.

During finger-tapping stimuli, the VA, VLa, VLp, VPL, and CM nuclei showed strong BOLD response. Our findings are consistent with previous findings (Ilinsky and Kultas-Ilinsky, 1987; Fang et al., 2006; Kumar et al., 2015) suggesting that the VA, VLa, and VLp nuclei are the main sources of projections to the motor cortical areas, while also additional motor-related intralaminar und medial nuclei (CM, Pf, and MD) were observed to send inputs to the primary motor areas and premotor areas. Interestingly, the motor nuclei presented in this study are also shown to be associated with the hand movement motor task (Kumar et al., 2022). Tactile stimuli evoked strong BOLD response in the VPL nucleus. Strong tactile activation was also detected in the VLp and CM nuclei. In line with a recent fMRI study in rats (Sanganahalli et al., 2022), our results indicate that the VPL nucleus plays an important role in the sensorimotor processing in humans, even during passive tactile stimulation.

In previous animal studies, the CM/PF thalamic complex was reported to be associated with attentional processing (Kinomura et al., 1996) and motor adjustment (Van der Werf et al., 2002). As a result of a neuroanatomical tracing analysis (Van der Werf et al., 2002), a distinction between Pf and CM functions was proposed, which assigned the CM nucleus with a specific involvement during sensorimotor functions and the Pf nucleus with a specific role during associative-limbic motor functions. This distinction in humans using fMRI studies is, however, not possible with our study setup. Nevertheless, our results also suggest the involvement of the CM nucleus in general attentional and sensorimotor processes.

The results of this work coincide with previous studies that have demonstrated an association between the pulvinar nuclei and attention/visual stimulus (Petersen et al., 1987; Fischer and Whitney, 2012; Zhou et al., 2016). Compared to the tactile task during which participants were asked to focus on a fixation cross, a higher pulvinar nuclei activation was found for the finger-tapping task, during which the participants viewed a blinking arrow along with the word “fingers” indicating the task. This may be further evidence of the association between the pulvinar nuclei and visual stimulus. However, in this case, the left pulvinar nuclei showed stronger activation, which may be related to a higher attention on the right hand performing the task.

### Limitations and future directions

Due to the complex and laborious experimental set-up along with scan time constraints, we applied the active and passive stimuli only to the fingers of the right hand in right-handed subjects. However, we expect that active and passive fMRI tasks can evoke the same activation pattern in the contralateral side (Nakamura et al., 2020). Therefore, it appears interesting to explore in future work whether stimulating the left hand in right handed subjects would yield comparable thalamic activation features in the right hemisphere. Despite the small number of participants, our study allowed us to identify the main thalamic nuclei involved in a sensorimotor task with visual as well as focusing elements, with the findings consistent over all participants. However, because only healthy subjects participated, there is no direct clinical-radiological correlation yet. A comparison with pre-surgical data from Parkinson’s disease patients eligible for deep brain stimulation (DBS) could be a potential follow-up step to establish whether functional MR image guidance may prove helpful to enable more accurate electrode placement.

Despite imaging at ultra-high field strength benefits from an enhancement of the SNR, which can be translated into an increased spatial resolution, precise anatomical segmentation based on structural ultra-high field data yet constitutes a major challenge. An increased spatial resolution may reveal more fine-grained structural details, such as small sulci and gyri, however, accurately detecting these nuances using currently available segmentation software such as FreeSurfer remains difficult. The altered contrast at ultra-high field strength, caused by differences in relaxation times as well as increased static and transmit field inhomogeneities compared to lower magnetic field strengths, results in more complex tissue contrast patterns, which cannot be handled properly by contemporary segmentation tools as those are optimized for image contrasts at lower fields. A segmentation based on structural 9.4T data would lead to inaccurate results and a cascade of errors in downstream analysis. Therefore, to segment individual thalamic nuclei, we used structural data acquired at 3T with an isotropic resolution of 0.8 mm, which was sufficiently high to resolve the anatomical structures in our 1.25 mm isotropic functional data acquired at 9.4T. The 3T segmentation results were deemed more accurate on visual inspection and successfully minimized any cascading effects.

In this study, we focused primarily on the functional localization of thalamic nuclei involved in sensorimotor processes. However, the imaging protocol was designed to cover the entire brain since in future work, we intend to investigate the functional connectivity between the individual thalamic nuclei and different cortical areas as well as basal ganglia. Furthermore, an improvement in future studies may involve the implementation of passive motor tasks in addition to active motor tasks as employed here. This might not only provide an interesting comparison of voluntary and non-voluntary movements, but could also avoid the strong relayed inputs from the cortex and basal ganglia. However, this requires a more complex setup to apply passive movements in an automatized manner.

To our knowledge, the present study is the first to identify the sensorimotor thalamic nuclei in humans using task-based fMRI at a field strength of 9.4T. Our data provide new insight into the functional localization of the individual thalamic nuclei, as well as further evidence for the important role of the thalamus in processing a variety of inputs. The ability to consistently reproduce such results in order to localize the individual thalamic nuclei is crucial to assess the feasibility of fMRI for pre-surgical mapping. By addressing the challenges arising from the highly variable size and shape of the thalamus across individuals and taking into account the inconsistency between common atlases, ultra-high field fMRI may have the potential to enable pre-surgical localization of thalamic nuclei in a more accurate manner tailored to each patient.

## Data availability statement

The raw data supporting the conclusions of this article will be made available by the authors, without undue reservation.

## Ethics statement

The studies involving human participants were reviewed and approved by Ethics Committee of University of Tübingen. The patients/participants provided their written informed consent to participate in this study.

## Author contributions

EC: study design, stimulus programming, data acquisition, planning and performing the data analysis, writing–original draft, and preparing the figures. RH: assistance in data acquisition, reviewing and editing the manuscript. ME: advice for data evaluation and reviewing the manuscript. FM: assistance in data acquisition and reviewing the manuscript. VK: reviewing and editing the manuscript. WG: supervision, reviewing and editing the manuscript. KS: resources, supervision, funding acquisition, reviewing and editing the manuscript. JB: advice for MR measurements and protocol optimization, reviewing and editing the manuscript. All authors contributed to the article and approved the submitted version.

## Footnotes

1
www.neurobs.com

2
http://www.fil.ion.ucl.ac.uk/spm

3
http://surfer.nmr.mgh.harvard.edu

## Supplementary material

Supplementary Figure 1 **(A)** Representative temporal SNR maps for GE EPI performed using an 1.25 mm isotropic resolution and a multiband factor of 2 (MB2) at 9.4T without (left) and with (right) NORDIC correction. **(B)** Thalamic activation (motor stimulus) obtained in a single subject (S5). T-maps (uncorrected *p* < 0.001) without (left) and with (right) NORDIC correction.

Supplementary Figure 2 Anatomical image quality and SPM co-registration results for one subject (S2). First, the 3T T2-weighted (T2w) image was co-registered to the 3T T1-weighted (T1w) image, which were then both co-registered to the 9.4T T1w image. A: Coronal **(top row)** and axial **(bottom row)** views of the 9.4T T1w (left), 3T T1w **(middle)**, and 3T T2w (right) whole-brain images. B: Illustration of the zoomed area marked with a blue box in A. The green and red lines indicate the image contours of the 3T T1w and 3T T2w images calculated by the CheckReg tool in SPM, respectively. Left column: contours of the co-registered 3T T1w (green) and T2w (red) images on the 9.4T T1w image. Middle column: contours of the co-registered 3T T2w image on the 3T T1w image. Right column: contours of the co-registered 3T T1w image on the 3T T2w image.

Supplementary Figure 3 Whole-brain single-subject GLM results. Panel **(A)** illustrates the task-evoked neural activity in the cerebral cortex for the motor (top row) and tactile (bottom row) task-based fMRI experiments in four representative subjects (S1, S3, S5, and S6). Similarly, panel **(B)** depicts the neural activity in the right cerebellum in response to the same tasks in the same four subjects. For each subject, the t-statistic maps (*p* < 0.05 FWE with a minimum cluster size of 20 voxels) are superimposed on the most representative axial (A) and sagittal (B) slices of a 3T T1-weighted MPRAGE image in neurological convention.

Supplementary Figure 4 Difference between motor and tactile task-based activation, calculated by subtracting the relative number of activated voxels (*p* < 0.001, uncorrected) in percentage of the tactile task (cf. Figure 5) from the respective value of the motor task (cf. Figure 3) within left **(A)** and right **(B)** thalamic nuclei. Please note that only the eight subjects, who participated in both tasks, are included here.
