# Supplementary figures and images for: Functional mapping of sensorimotor activation in the human thalamus at 9.4 Tesla
Source: Front Neurosci. 2023 Mar 15;17:1116002. doi: 10.3389/fnins.2023.1116002 (PMC10050447; doi:10.3389/fnins.2023.1116002)

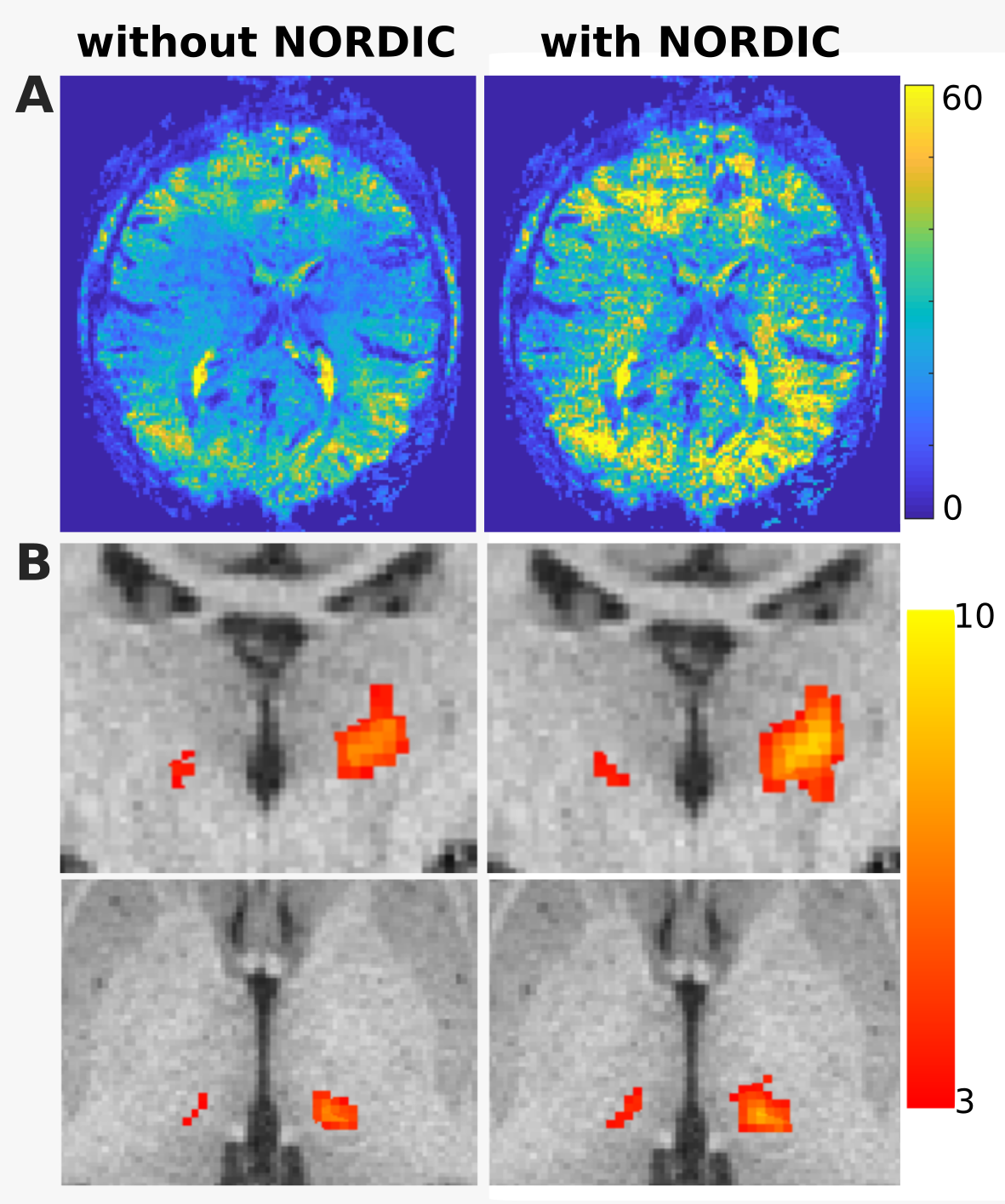

Supplement: Supplementary Figure 1 — (A) Representative temporal SNR maps for GE EPI performed using an 1.25 mm isotropic resolution and a multiband factor of 2 (MB2) at 9.4T without (left) and with (right) NORDIC correction. (B) Thalamic activation (motor stimulus) obtained in a single subject (S5). T-maps (uncorrected p < 0.001) without (left) and with (right) NORDIC correction. [file Image_1.TIF]

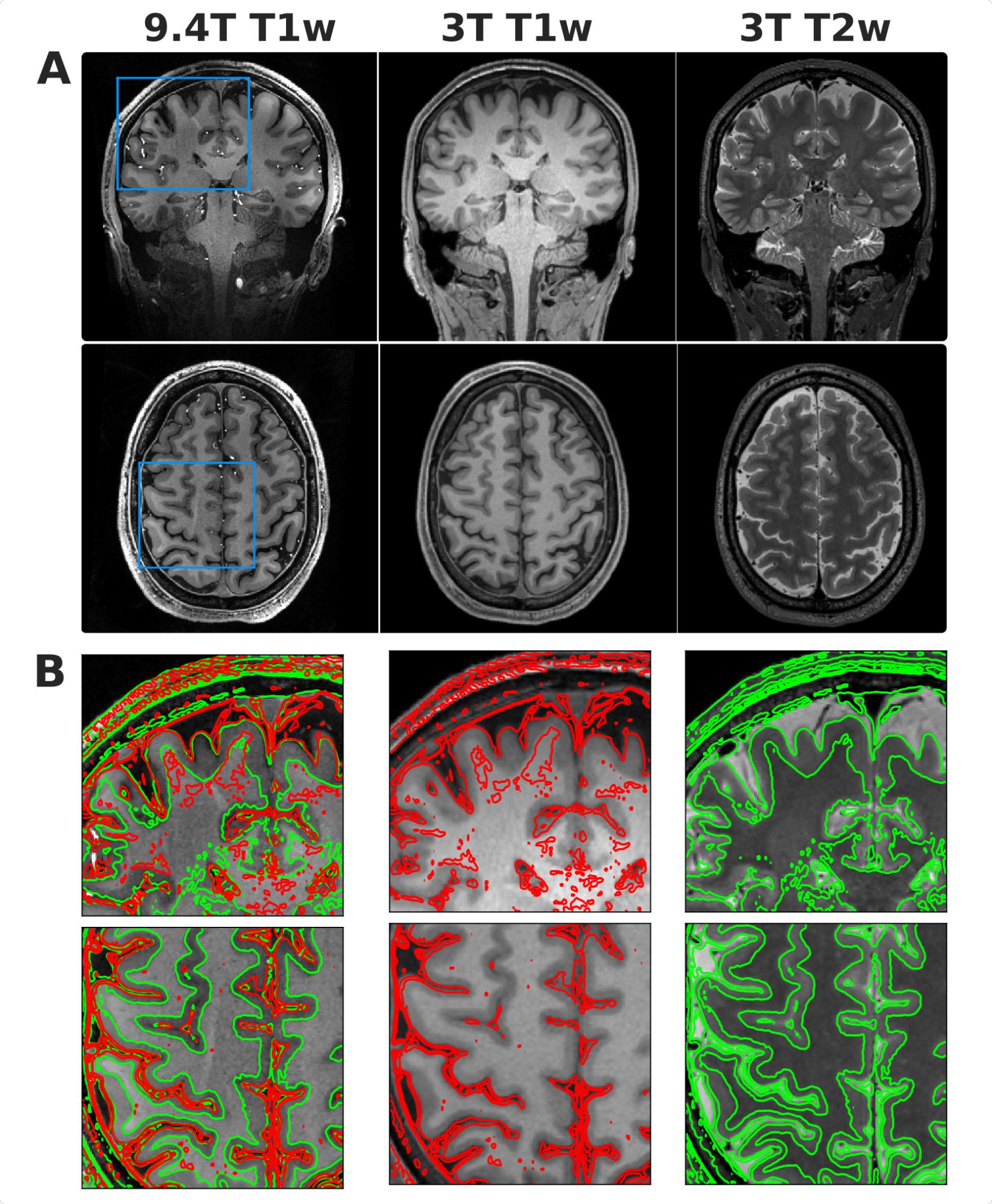

Supplement: Supplementary Figure 2 — Anatomical image quality and SPM co-registration results for one subject (S2). First, the 3T T2-weighted (T2w) image was co-registered to the 3T T1-weighted (T1w) image, which were then both co-registered to the 9.4T T1w image. A: Coronal (top row) and axial (bottom row) views of the 9.4T T1w (left), 3T T1w (middle), and 3T T2w (right) whole-brain images. B: Illustration of the zoomed area marked with a blue box in A. The green and red lines indicate the image contours of the 3T T1w and 3T T2w images calculated by the CheckReg tool in SPM, respectively. Left column: contours of the co-registered 3T T1w (green) and T2w (red) images on the 9.4T T1w image. Middle column: contours of the co-registered 3T T2w image on the 3T T1w image. Right column: contours of the co-registered 3T T1w image on the 3T T2w image. [file Image_2.TIF]

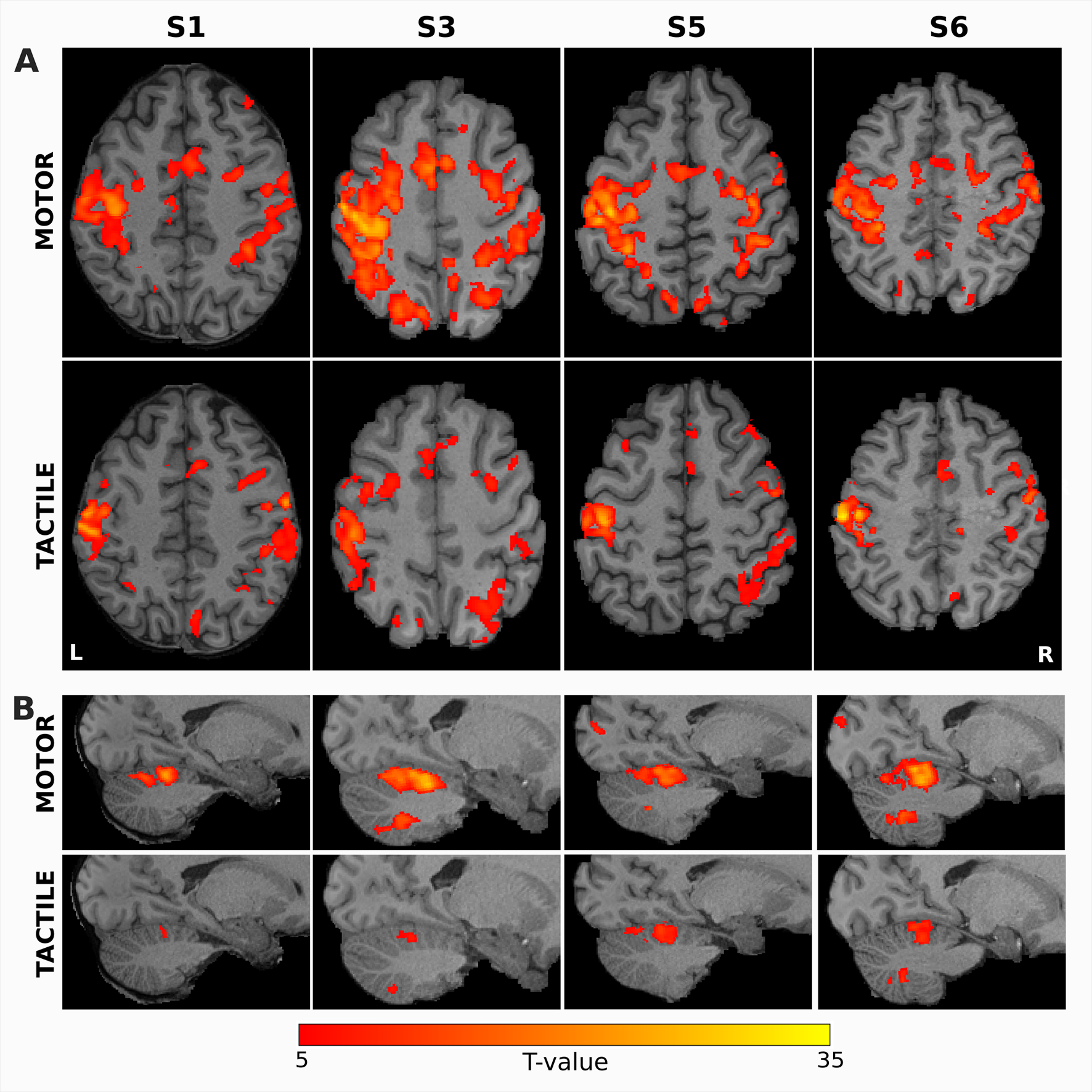

Supplement: Supplementary Figure 3 — Whole-brain single-subject GLM results. Panel (A) illustrates the task-evoked neural activity in the cerebral cortex for the motor (top row) and tactile (bottom row) task-based fMRI experiments in four representative subjects (S1, S3, S5, and S6). Similarly, panel (B) depicts the neural activity in the right cerebellum in response to the same tasks in the same four subjects. For each subject, the t-statistic maps (p < 0.05 FWE with a minimum cluster size of 20 voxels) are superimposed on the most representative axial (A) and sagittal (B) slices of a 3T T1-weighted MPRAGE image in neurological convention. [file Image_3.TIFF]

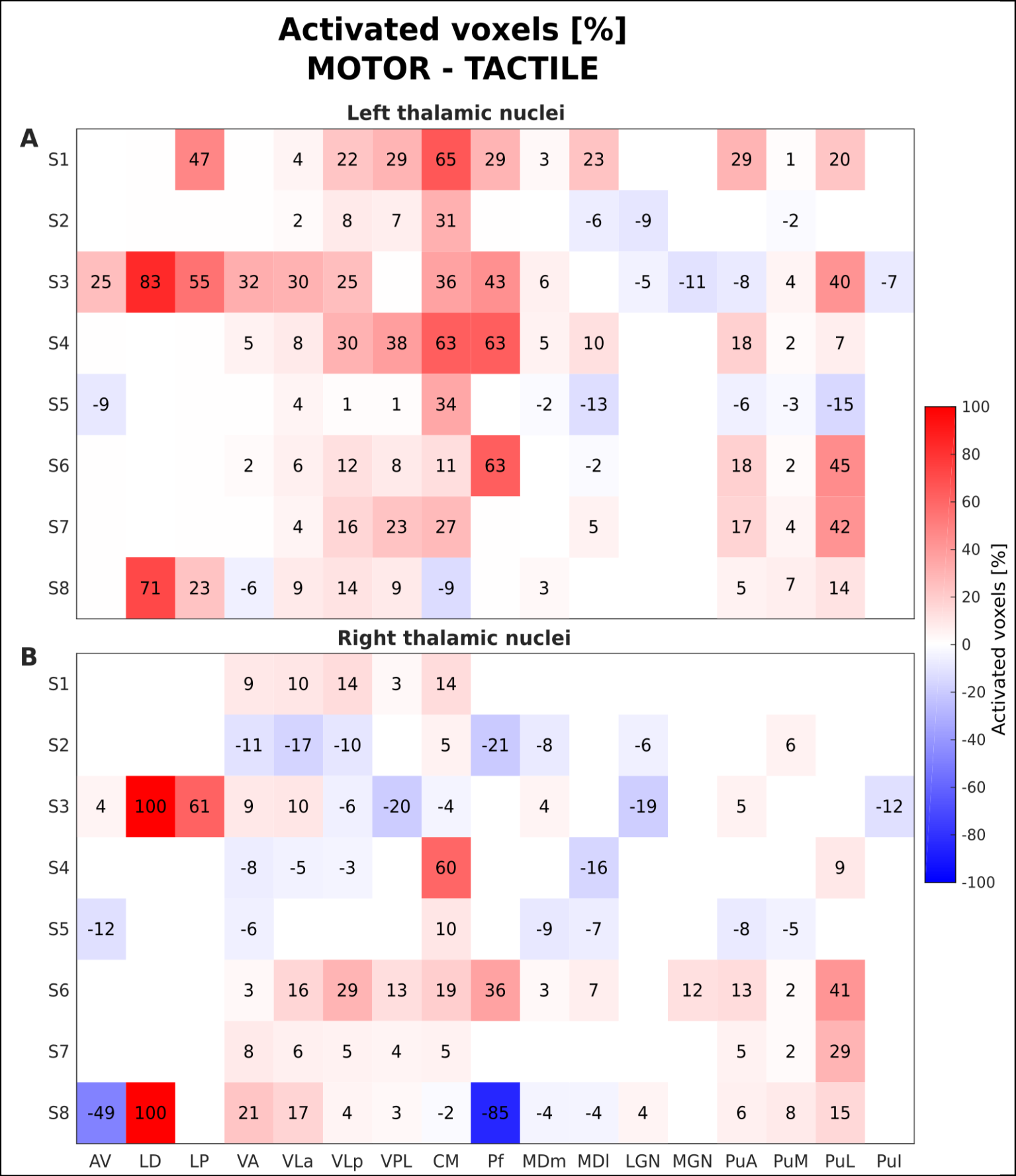

Supplement: Supplementary Figure 4 — Difference between motor and tactile task-based activation, calculated by subtracting the relative number of activated voxels (p < 0.001, uncorrected) in percentage of the tactile task (cf. Figure 5) from the respective value of the motor task (cf. Figure 3) within left (A) and right (B) thalamic nuclei. Please note that only the eight subjects, who participated in both tasks, are included here. [file Image_4.TIF]
